# Zika vaccine candidates progress through nonclinical development and enter clinical trials

**DOI:** 10.1038/npjvaccines.2016.23

**Published:** 2016-11-10

**Authors:** Alan D T Barrett

**Affiliations:** 1 Department of Pathology and Sealy Center for Vaccine Development, University of Texas Medical Branch, Galveston, TX, USA

Zika virus (ZIKV), a mosquito-borne flavivirus, was first identified in the 1940s in Africa, emerged in the Americas in Brazil in May 2015, with evidence that supports a causal role for this virus in a range of clinical disease presentations, collectively termed Zika congenital syndrome (ZCS), including identification of the virus in the brains of children who were still-born with microcephaly. Subsequently, the virus rapidly spread through the Americas with at least 47 countries and territories reporting clinical cases. As one might expect, many research groups in government, academia, industry, and non-governmental organizations have applied their expertise to investigating ZIKV. Prior to 2015, there had been little research on ZIKV as it was not considered to be a major pathogen and there was little existing expertise on the virus. Extraordinary progress has been made over the last year with nearly 1000 publications reporting investigations on the virus and the different clinical syndromes, demonstrating not only the excellent toolbox available to scientists today but also the speed with which multi-authors/multi-institutions can work together.

Much effort has been devoted to Zika vaccine development with at least 40 entities working on developing vaccine candidates using a variety of platforms, which have been reviewed in recent papers.^1–11^ In addition, to help vaccine developers, the World Health Organization published a Target Product Profile (TPP) for a Zika vaccine to be used in an emergency scenario (http://www.who.int/immunization/research/meetings_workshops/WHO_Zika_vaccine_TPP.pdf?ua=1) and a website for the vaccine pipeline tracker (http://www.who.int/immunization/research/vaccine_pipeline_tracker_spreadsheet/en/).

In the last 3 months, the first papers have been published describing nonclinical studies with candidate Zika vaccines in animal models^12–16^ to investigate protective immunity and passive protection, including the latest published in *npj Vaccines* by Muthumani *et al.*^16^ These five papers describe utilization of four platforms: purified formalin inactivated virus adjuvanted with alum (PIV), recombinant envelope (rE) protein, three different plasmid DNA constructs that all encode the ZIKV prM/E genes, and two adenovirus vectors expressing ZIKV prM/E or rE genes. All platforms induced protective immunity and passive protection in mice and non-human primates (NHPs) with at least one of the regimens investigated.

Larocca *et al.*^12^ demonstrated induction of protective immunity with a single dose of their 50 μg DNA vaccine based on a Brazilian ZIKV sequence given by the IM) route in Balb/c, SJ/L, and C57BL/6 mouse models with undetectable viremia following virus challenge (either homologous Brazilian or heterologous Puerto Rico strains by the intravenous route) at 4 or 8 weeks post immunization. Protection was correlated with the induction of neutralizing antibodies, and although CD4^+^ and CD8^+^ lymphocyte responses were induced, they were not required for protection. This group also compared their DNA vaccine with a single dose of 1 μg PIV based on the Puerto Rico strain given by the IM and SC routes. The PIV given by the IM route was superior to that given by the SC route. They followed up their mouse studies with a study in rhesus macaques. Here Abbink *et al.*^13^ compared the DNA and PIV candidate vaccines with a Rhesus adenovirus serotype 52 (RhAd52) vector-based vaccine. The PIV was given as a two-dose regimen of 5 μg PIV at 0 and 4 weeks by the SC route. The DNA vaccine was also given as a two-dose 5 mg DNA regimen at 0 and 4 weeks by the IM route, while the RhAD52 vaccine was given as a single dose of 10^11^ virus particle dose by the IM route. The three different platforms induced protective immunity in NHPs, including neutralizing antibodies and protection from challenge with 10^3^ p.f.u. (plaque-forming unit) of a Brazilian strain by the SC route as measured by undetectable viremia (<100 copies/ml by qRT–PCR), and absence of detection of viral RNA in tissues that were collected in some experiments. Furthermore, neutralization titers increased post challenge, suggesting the vaccine did not induce sterilizing immunity. Interestingly, while the DNA and PIV vaccines appeared comparable in the mouse models, the PIV was superior in these limited NHP studies. Also, the RhAd52 vaccine was strongly immunogenic after one dose, while the other two vaccine platforms needed two doses.

Kim *et al.*^14^ compared immunity induced in C57BL/6 mice by a two-dose (0 and 2 weeks) regimen of either 20 μg codon-optimized recombinant E protein ectodomain of a Brazilian strain fused to the T4 fibritin foldon trimerization domain (Efl) to 10^11^ virus particles of the same construct on an adenovirus 5 vector (Ad5.ZIKV-Efl) following intradermal (ID) and primary SC with intranasal booster administration, respectively. Both constructs induced neutralizing antibodies, although the Efl protein induced lower neutralization titers than Ad5.ZIKV-Efl. Immunization of the Ad5.ZIKV-Efl vaccine candidate, but not the Efl candidate, resulted in passive protection in 7-day-old offspring following ip challenge with 10^5^ pfu of ZIKV African strain Dak Ar 41542.

Dowd *et al.*^15^ utilized both mouse and rhesus macaque models using a DNA vaccine based on the French Polynesia strain. These authors used a single dose of either 2, 10, or 50 μg of DNA in Balb/c and C57BL/6 mouse models, and all immunogens induced similar levels of neutralizing antibodies (note this study did not look at the cellular immune response). For the NHP studies, this group used 1 and 4 mg per animal in a two-dose regimen (0 and 4 weeks) and compared with a single-dose regimen of 1 mg per animal. The two-dose regimen was superior to the one-dose regimen in terms of induction of neutralizing antibodies. All NHPs were challenged at 8 weeks post dose-one of vaccine with 10^3 ^ffu of the Puerto Rico strain by the SC route. The one-dose vaccine was not protective while 17 of 18 animals given the two-dose regimen had no detectable viremia by qRT–PCR.

The most recent report is by Muthumani *et al.*^16^ A three-dose regimen (0, 2 and 4 weeks) of a 25 μg synthetic consensus ZIKV prM/E sequence DNA vaccine was found to induce both cellular and humoral immunity in mouse models. Significantly, in addition to immunocompetent C57BL/6 mice, these authors immunized immunocompromised mice lacking interferon αβ receptors (IFNAR^−/−^) mice, which is a lethal challenge model as compared with the standard viremia challenge model in immunocompetent mice. IFNAR^−/−^ mice were immunized with either a one or a two 25 μg dose regimen (0 and 2 weeks) and challenged with 10^6^ pfu Puerto Rico strain by the SC route. Both vaccination regimens protected the mice from weight loss and death. Subsequently, rhesus macaques were immunized by the ID route with 2 mg/dose in a two-dose regimen given at 0 and 4 weeks. Again, the vaccine induced a robust cellular and humoral immune response, including neutralizing antibodies.

Passive protection studies have been used for licensed flavivirus vaccines as part of the approval process, for example, the inactivated Japanese encephalitis vaccine.^17^ Here animals are passively administered with antibody (monoclonal antibodies or sera from immunized animals or humans) prior to challenge with wild-type virus. In the case of the candidate Zika vaccines, the studies describe a range of neutralization titers that are required to mediate passive protection. However, these animal studies are consistent with induction of neutralizing antibodies being a potential surrogate of protection, although the protective titer is unclear.

It is important to realize that the different studies cannot be directly compared with each other, as the methodology to measure neutralizing antibodies is different between studies with a combination of focus reduction neutralization test (FRNT), plaque reduction neutralization test (PRNT), micro-neutralization (MN), and/or reporter virus particle (RVP) assays being used. In addition, there are no antibody standards available yet with which to standardize the different neutralization methods. Nonetheless, based on passive protection studies, all five studies suggest that a neutralization titer of at least 100 is likely to be associated with a protective immune response. Interestingly, if correct, this is higher than that for current licensed flavivirus vaccines. For Japanese encephalitis (formalin inactivated, live attenuated or recombinant chimeric live attenuated), and tick-borne encephalitis (formalin inactivated) vaccines a neutralization titer of 1 in 10 is considered protective, while for yellow fever (live attenuated), a neutralization titer of 1 in 10–50 is protective. So what is different about ZIKV? One possibility to consider is that ZIKV is harder to neutralize by antibodies as compared with other flaviviruses. Basic science results support this hypothesis. Structural studies suggest that ZIKV is more stable than some other mosquito-borne flaviviruses studied,^18,19^ although this may be a variable phenomenon, as suggested by the recent publication by Goo *et al.*.^20^ Also, panels of mouse and human monoclonal antibodies recently described neutralize ZIKV poorly compared with similar antibodies raised against dengue and West Nile viruses.^21–24^ Thus, basic science studies appear to be consistent with applied studies with vaccine candidates. It is possible that future basic science studies will elucidate approaches to improve the ability of immunogens to stimulate antibodies with higher affinity for ZIKV epitopes critical for eliciting neutralizing antibodies.

Overall, the nonclinical studies are consistent with induction of neutralizing antibodies being a potential surrogate of protection, but this would need substantiating with clinical studies involving immunogenicity and clinical endpoint(s) for protection, as results from animals do not always translate to the situation in humans. Studies on the durability of the vaccine-induced immune response are also limited. However, it is encouraging that the candidate vaccines induce immunity that results in undetectable viremia as measured by qRT–PCR post challenge, but the very limited data suggest that, like the currently licensed flavivirus vaccines, these candidate ZIKV vaccines may not induce sterilizing immunity. Some of the vaccine candidates reported in the nonclinical papers have advanced into clinical evaluation (NCT02840487, NCT02887482, and NCT02809443) and it will be very interesting to see how the candidate vaccines perform in immunogenicity studies.
